# Development and Evaluation of SoySNP50K, a High-Density Genotyping Array for Soybean

**DOI:** 10.1371/journal.pone.0054985

**Published:** 2013-01-25

**Authors:** Qijian Song, David L. Hyten, Gaofeng Jia, Charles V. Quigley, Edward W. Fickus, Randall L. Nelson, Perry B. Cregan

**Affiliations:** 1 Soybean Genomics and Improvement Laboratory, Agricultural Research Service, United States Department of Agriculture, Beltsville, Maryland, United States of America; 2 Pathology and Genetics Research Unit and Department of Crop Sciences, Soybean/Maize Germplasm, Agricultural Research Service, United States Department of Agriculture, University of Illinois, Urbana, Illinois, United States of America; Nanjing Agricultural University, China

## Abstract

The objective of this research was to identify single nucleotide polymorphisms (SNPs) and to develop an Illumina Infinium BeadChip that contained over 50,000 SNPs from soybean (*Glycine max* L. Merr.). A total of 498,921,777 reads 35–45****bp in length were obtained from DNA sequence analysis of reduced representation libraries from several soybean accessions which included six cultivated and two wild soybean (*G. soja* Sieb. et Zucc.) genotypes. These reads were mapped to the soybean whole genome sequence and 209,903 SNPs were identified. After applying several filters, a total of 146,161 of the 209,903 SNPs were determined to be ideal candidates for Illumina Infinium II BeadChip design. To equalize the distance between selected SNPs, increase assay success rate, and minimize the number of SNPs with low minor allele frequency, an iteration algorithm based on a selection index was developed and used to select 60,800 SNPs for Infinium BeadChip design. Of the 60,800 SNPs, 50,701 were targeted to euchromatic regions and 10,000 to heterochromatic regions of the 20 soybean chromosomes. In addition, 99 SNPs were targeted to unanchored sequence scaffolds. Of the 60,800 SNPs, a total of 52,041 passed Illumina’s manufacturing phase to produce the SoySNP50K iSelect BeadChip. Validation of the SoySNP50K chip with 96 landrace genotypes, 96 elite cultivars and 96 wild soybean accessions showed that 47,337 SNPs were polymorphic and generated successful SNP allele calls. In addition, 40,841 of the 47,337 SNPs (86%) had minor allele frequencies ≥10% among the landraces, elite cultivars and the wild soybean accessions. A total of 620 and 42 candidate regions which may be associated with domestication and recent selection were identified, respectively. The SoySNP50K iSelect SNP beadchip will be a powerful tool for characterizing soybean genetic diversity and linkage disequilibrium, and for constructing high resolution linkage maps to improve the soybean whole genome sequence assembly.

## Introduction

Even with the extensive linkage disequilibrium (LD) in the soybean [*Glycine max* (L). Merr.] genome, a large number of molecular markers are required to cover the genome for the purpose of genome-wide genetic analysis. Single nucleotide polymorphisms (SNPs) are the most abundant form of DNA polymorphism in eukaryotic genomes [1], and are suitable for the development of high-throughput, easy-to-automate genotyping methods because most SNPs have only two alleles, thereby simplifying genotyping approaches and analysis [2]–[6]. Previous procedures of SNP discovery in plants involved primer selection, PCR amplification and sequencing of amplicons from a small set of diverse genotypes [7]–[9]. This approach is time-consuming and expensive. With the advent of second generation sequencing technology, large amounts of DNA sequence data can be generated in a short period of time. Coupled with the availability of the soybean whole genome sequence [10], large numbers of sequence variants can be efficiently identified via the alignment of the short sequence reads from diverse genotypes to the whole genome sequence [11]–[13]. High throughput SNP discovery is a necessity in light of the availability of high throughput SNP assay systems such as the Infinium platform from Illumina that allows the assay of large numbers of SNPs per DNA sample in parallel on a single silicon slide (http://www.illumina.com/). Thus, the objective of this research was to efficiently identify large numbers of SNPs in soybean using advanced sequencing technologies and SNP identification pipelines; to develop a custom high resolution Illumina Infinium BeadChip containing SNPs with high minor allele frequency, even distribution across the euchromatic and heterochromatic regions of the soybean genome; to evaluate the performance of the chip in a set of diverse landrace and elite soybean genotypes as well as in a diverse set of wild soybean [*G. soja* (Sieb. et Zucc.)] genotypes and to identify candidate regions that may be associated with domestication and recent selection imposed by plant breeding.

## Results

### SNP Discovery and Randomness of SNP Distribution in the Soybean Genome

The total number of short reads and total bases obtained from the eight genotypes were 498,921,777 and 18,313 Mb, respectively and the total bases obtained from the eight genotypes ranged from 277 Mb (0.3×) to 6,473 Mb (6.8×) (Table 1). Following alignment of the short reads to the Williams 82 (Glyma1.01) genome sequence, 209,903 SNPs were identified by MAQ and/or CASAVA. The average distance between adjacent SNPs was 4.5 kb. Only 18, 193 and 1575 of the 209,863 gaps between adjacent SNPs had sequence length >200 kb, 100 kb and 50 kb, respectively and the largest gap was 328 kb. Thus, in general, the identified SNPs were fairly well distributed throughout the soybean genome.

**Table 1:** Illumina GAII DNA sequence analysis of eight genotypes and mixed DNA.

Genotypes	Number of lanes	Number of reads	Total number of bases
PI 468916 and PI 479752	23	120,581,402	4,258,255,310
Essex	18	166,829,261	6,473,293,963
Evans	4	11,638,328	360,108,084
Archer	3	7,759,202	277,664,148
Minsoy	3	26,101,751	881,054,603
Noir 1	3	39,234,960	1,404,545,136
Peking	2	35,012,819	1,237,429,040
Mixed DNA	15	91,764,054	3,421,615,732
Total	71	498,921,777	18,313,966,016

### SNP Filtering

Of the 209,903 SNPs, a total of 28,891 were A vs. T or G vs. C SNPs and thus, the remaining 188,272 SNPs were a potential source for Infinium II assay (duel color channel, single bead). After eliminating SNPs with N’s or ambiguity codes in the 60 nt of flanking sequence and SNPs residing within 25 nt of another SNP, a total of 150,171 of the 188,272 SNPs for Infinium II assay remained. After elimination of the SNPs with flanking sequences that were not unique in the genome, a total of 146,161 SNPs, including 785 SNPs from unanchored scaffolds, was retained and these SNPs were ideal candidates for Infinium II assay design.

### Initial SNP Verification and Calculation of Priority Scores

Of the 146,161 SNPs, a total of 23,721, 50,229, 58,281 and 13,930 were assigned to priority groups of A, B, C, and D, respectively. Initial validation of 767 SNPs via Sanger sequencing gave validation rates of 100%, 83%, 77% and 67% for the SNPs with the A, B, C, and D scores, respectively (Table 2). These rates were used as priority scores (R_i_) for the corresponding priority groups for calculation of the SNP selection index.

**Table 2:** Validation rate of SNPs based on the Sanger sequence analysis of a random set of 767 SNP loci.

Priority class	Total numberof loci	Number of loci with null or multiple amplicons	Number of lociwith good sequence	Number of SNPs validated	Number of SNPsnot validated	Validationrate (%)
A	89	17	72	72	0	100
B	233	43	190	158	32	83
C	390	73	317	244	73	77
D	55	9	46	31	15	67

### Selection of a Set of 60,800 SNPs

Because Illumina only allowed 60,800 SNPs in the bead pool at the time the chip was developed, a total of 60,701 SNPs were selected from the 20 chromosomes (Gm01–Gm20) and 99 SNPs were selected from 71 unanchored sequence scaffolds (Table S1). Of the 60,701 SNPs, a total of 50,701were selected from the euchromatic regions and 10,000 SNPs were selected from heterochromatic regions with approximately a 5∶1 ratio. Thus, the average density of SNPs was approximately 110.5 SNPs per Mb and 20.4 SNPs per Mb in euchromatic and heterochromatic regions, respectively (Table 3). The number of SNPs chosen from each chromosome was the product of the chromosome sequence length by the average SNP density in the euchromatic and heterochromatic regions, respectively. The SNPs along each chromosome were then selected with the iteration algorithm. Genetic mapping of the 99 SNPs from the 71 unanchored sequence scaffolds is expected to anchor 6.5 Mb of the 23 Mb of sequence that was not anchored in the Glyma1.01 build.

**Table 3:** Number and density of selected SNPs and the 146,161 SNPs in euchromatic and heterochromatic regions of each soybean chromosome.

Chromosome	Numberof SNPs	Number of selected SNPs in the euchromatic regions	Number ofselected SNPs in the heterochro-matic region	Sequence length of euchromatic regions(bp)	Sequencelength ofheterochroma-ticregions (bp)	SNP density in euchromaticregions (SNPs/Mb)	SNP density in the heterochro-matic regions (SNPs/Mb)	Number of SNPs of the 146,161 in the euchro-matic regions	Number of SNPs of the 146,161 in the heterochro-matic regions
Gm1	2489	1652	837	14841727	41073868	111.3	20.4	2688	5367
Gm2	3445	2929	516	26316426	25340287	111.3	20.4	4099	2852
Gm3	2691	2102	589	18879713	28901363	111.3	20.4	3245	4276
Gm4	2718	2099	619	18855914	30387938	111.3	20.4	3197	5888
Gm5	2927	2537	390	22797076	19139428	111.3	20.4	3062	1286
Gm6	3041	2458	583	22083366	28639455	111.3	20.4	2908	4851
Gm7	3421	3073	348	27609531	17073626	111.3	20.4	4586	1424
Gm8	3795	3473	322	31208512	15787020	111.3	20.4	4010	2062
Gm9	2556	1960	596	17602854	29240896	111.3	20.4	2828	5739
Gm10	3241	2696	545	24219274	26750361	111.3	20.4	3435	4328
Gm11	2610	2308	302	24367505	14805285	94.7	20.4	2441	1081
Gm12	2378	1910	468	17140105	22973035	111.3	20.4	2516	2340
Gm13	3591	3288	303	29558651	14850320	111.3	20.4	4286	2517
Gm14	2863	2265	598	20344958	29366246	111.3	20.4	3304	3014
Gm15	3164	2603	561	23378504	27560656	111.3	20.4	4398	5224
Gm16	2370	1969	401	17708632	19688753	111.3	20.4	3042	3155
Gm17	2694	2253	441	20240737	21666037	111.3	20.4	2957	4228
Gm18	4618	4099	519	36632197	25675943	111.3	20.4	8814	4680
Gm19	3520	3047	473	27373488	23215953	111.3	20.4	4371	4414
Gm20	2569	1980	589	17784173	28988994	111.3	20.4	2897	3546
Total	60701	50701	10000	4.59E+08	4.91E+08			73084	72272

### Index Score and Evenness of Spacing between the 60,800 Selected SNPs

**Figure 1:**
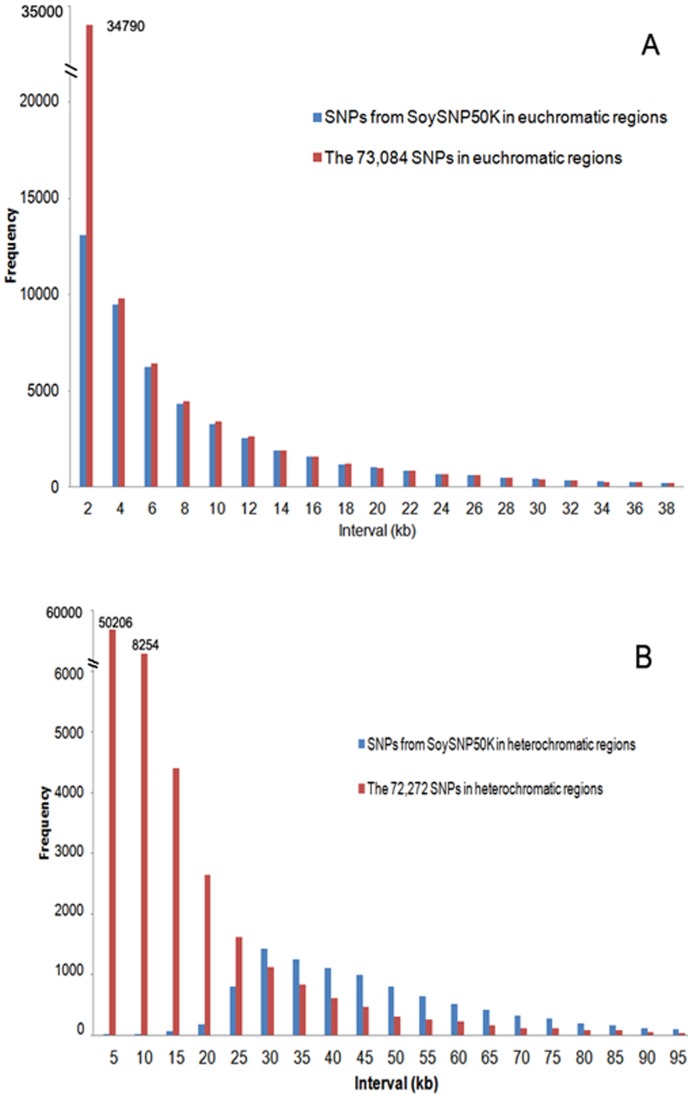
Distribution of distances between adjacent pre-selected and selected SNPs in euchromatic and heterochromatic regions. (A) Selected SNPs (50,701) in euchromatic regions of SoySNP50K and the pre-selected SNPs (73,084) in euchromatic regions. (B) Selected SNPs (10,000) in heterochromatic regions and pre-selected SNPs (72,272) in heterochromatic regions.

After SNP selection, the SNP selection index score of the 60,800 selected SNPs was increased versus the source set of 146,161 SNPs. The average SNP selection index score of the selected SNPs was 0.69 vs. 0.60 for the complete set of 146,161 SNPs. Of the 146,161 SNPs, 73,084 were located in euchromatic DNA and 72,272 were located in heterochromatic regions. A total of 50,701 SNPs were selected from the euchromatic regions and 10,000 were selected from the heterochromatic regions (Table 3). In the pre-selected set, the average distance between SNPs was 6.3 kb and 6.8 kb with a standard deviation of 10.7 kb and 14.9 kb in the euchromatic and heterochromatic regions, respectively. Of the 73,084 SNPs identified in the euchromatic regions, 21,721 (30%) were at distances of ≤2 kb between adjacent SNPs. In the heterochromatic regions, a total of 58,766 SNPs were at distances of ≤10 kb, which was 81% of the 72,272 SNPs in the heterochromatic regions. Of these, 62,272 were eliminated via the application of the iteration algorithm. After elimination of SNPs via the application of the iteration algorithm, the result was an average distance between adjacent SNPs in the selected set of 60,800 SNPs of 9.1 kb and 49.1 kb with a standard deviation of 9.1 kb and 33.3 kb in the euchromatic and heterochromatic regions, respectively. Thus, the uniformity of the distance between selected SNPs was enhanced in the euchromatic regions and clustered SNPs were greatly reduced in the heterochromatic regions as a result of SNP selection via the application of the iteration algorithm (Fig. 1).

### Distribution of SNPs in Genic Regions

A total of 16,381 (27%) of the 60,800 SNPs resided in genes and, of these, 6,820 SNPs were located in coding DNA sequence, 8,103 SNPs in introns, 752 SNPs in 5′untranslated regions and 706 SNPs in 3′UTR (Table S1). Within 2 kb upstream of genes there were a total of 7,707 SNPs and 6,723 SNPs were within 2 kb downstream of genes. Overall, more than 50% of the 60,800 SNPs resided within genes or within 2 kb upstream or downstream of genes.

### Infinium Chip Performance

Of the 60,800 SNPs submitted to Illumina for inclusion in the SNP assay, 52,041 SNPs, or 86% of the 60,800 passed Illumina manufacturing quality control and were included in the final bead pool of the SoySNP50K Infinium BeadChip. Validation of the SoySNP50K Infinium BeadChip was performed via the analysis of diverse germplasm including 96 landraces, 96 elite cultivars and 96 wild soybean accessions. Of the 52,041 SNPs, 47,337 (91%) were polymorphic and yielded successful SNP allele calls based upon clear separation between the fluorescent signals from the two homozygous genotypic classes for a given SNP as assessed with the normalized theta analysis of the Illumina GenomeStudio SNP analysis software.

The rate of SNPs producing successful SNP allele calls was associated with the SNP selection index score, the Illumina Infinium SNP design score and the priority group (Table 4). The correlation coefficients of the SNP selection index score and SNP score vs. the rate of SNPs with successful allele calls were 0.93 and 0.94, respectively, and both were significant at the 1% level. Correlations with priority group were not calculated because priority group is not a continuous variable and the number of priority groups is small. However, it appeared that higher priority group resulted in an increased rate of SNPs with successful allele calls (Table 4).

**Table 4:** Association of SNP selection index score, Illumina Infinium design score and priority group with the rate of SNPs with successful allele calls.

SNP selectionindex score (I_i_)	Number of SNPs with success-ful allele calls	Number of SNPsnot called	Rate of SNPs withsuccess-fulallele calls	Illumina Infinium design score (D_i_)	Number of SNPs with success-ful allele calls	Number of SNPsnot called	Rate of SNPswith success-fulallele calls	Priority group	Number of SNPswith success-fulallele calls	Number of SNPsnot called	Rate of SNPs with success-ful allele calls
≥0.9	5,974	210	0.97	≥0.9	22,359	1,326	0.94	A	10,943	582	0.95
≥0.8 & <0.9	7,630	410	0.95	≥0.8 & <0.9	10,316	800	0.93	B	16,163	1,536	0.91
≥0.7 & <0.8	10,995	749	0.94	≥0.7 & <0.8	5,755	605	0.90	C	16,505	2,003	0.89
≥0.6 & <0.7	11,102	1,000	0.92	≥0.6 & <0.7	3,649	460	0.89	D	3,835	474	0.89
≥0.5 & <0.6	5,507	663	0.89	≥0.5 & <0.6	2,414	458	0.84				
≥0.4 & <0.5	3,382	636	0.84	≥0.4 & <0.5	2,953	946	0.76				
<0.4	2,856	927	0.75								
Total	47,446	4,595			47,446	4,595			47,446	4,595	

### Estimate of SNP Minor Allele Frequency in Diverse Germplasm

Of the 47,337 SNP assays that produced successful allele calls, 40,841 (86%) had a minor allele frequency ≥10% when tested on the 96 landraces, 96 elite cultivars and 96 wild soybeans. Within the individual groups, the landraces had the most SNPs with a minor allele frequency ≥10% while the elite cultivars had the fewest SNPs with a minor allele frequency ≥10% (Table 5). Only 3,294 of the 47,337 had a minor allele frequency ≤5% within the diverse germplasm (Table 5).

**Table 5:** Distribution of SNP minor allele frequency in elite cultivar, landrace and wild soybean populations.

Minor allele frequency	Elite population	Landrace population	Wild population	All 288 genotypes
<0.05	13,114(27.9%)	8,060(17.1%)	8,833(19.3%)	3,294(7.0%)
≥0.05 & <0.1	3,358 (7.1%)	4,432(9.4%)	5,186(11.3%)	3,202(6.8%)
≥0.1 & <0.2	6,377 (13.6%)	8,813(18.8%)	9,300(20.3%)	7,837(16.6%)
≥0.2 & <0.3	7,665(16.3%)	8,966(19.1%)	7,913(17.3%)	10,016(21.2%)
≥0.3 & <0.4	8,393(17.9%)	8,511(18.1%)	7,094(15.5%)	10,890(23.0%)
≥0.4 & ≤0.5	8,085(17.2%)	8,210(17.5%)	7,464(16.3%)	11,999(25.3%)
Total	46992	46992	45,790	47,337

### Estimate of Genetic Distance and Number of Polymorphic SNPs among Pairs of Genotypes within the 96 Landraces, the 96 Elite Cultivars and the 96 Wild Accessions

Genetic distances of 47.6% of the pairs of genotypes among the elite cultivars, 68.1% among the landraces and 48.1% among the wild accessions were greater than 0.3 based on the SNPs with minor allele frequency (MAF) >0.05 (Table 6). The mean genetic distance and the standard deviation were 0.294 and 0.0393 among pairs of elite cultivars, 0.322 and 0.0518 among pairs of landraces, and 0.292 and 0.0528 among pairs of wild accessions, respectively. On average, 9,974 and 18,151 SNPs were expected to be polymorphic between a random pair of elite cultivars or landraces, respectively. A total of 9,094 to15,565, 9,240 to 17,761 and 7,889 to 16,576 SNPs were projected to be polymorphic between any randomly selected pair of elite cultivars, landraces and wild soybean accessions at the 95% probability level, respectively.

**Table 6:** Distribution of genetic distance based upon the proportion of polymorphic SNPs between pairs of elite cultivar, landrace and wild soybean genotypes.

Genetic distance	Number of pairs among elite genotypes	Number of pairs among landrace genotypes	Number of pairs among wild genotypes
<0.1	1 (0.0%)	4 (0.1%)	51 (1.1%)
≥0.1 & <0.2	68 (1.5%)	71 (1.6%)	202(4.4%)
≥0.2 & <0.3	2,319 (50.9%)	1,377 (30.2%)	2,120 (46.5%)
≥0.3 & <0.4	2,172 (47.6%)	2,861 (62.7%)	2,167 (47.5%)
≥0.4 & ≤0.5	0 (0.0%)	247 (5.4%)	20 (0.4%)
Total	4,560	4,560	4,560

### Identification of Regions with Signatures of Selection

**Figure 2:**
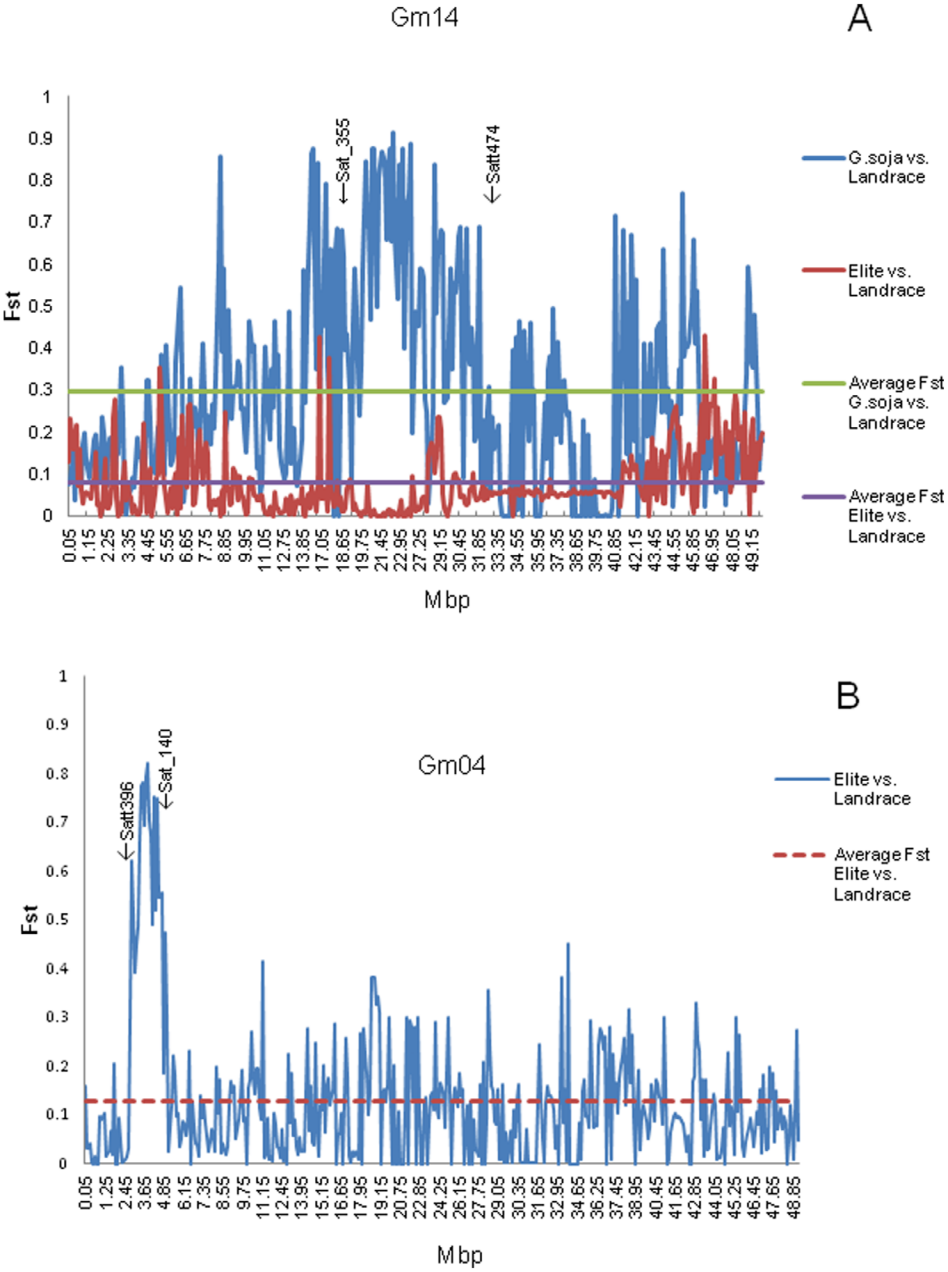
F_st_ values of *G. soja* vs. landraces and elite cultivars vs. landraces along chromosome Gm14 and Gm04. (A) Significant F_st_ values on Gm14 between SSR markers Sat_355 and Satt474 may contain loci associated with soybean domestication (B) Significant F_st_ values between SSR markers Satt396 and Sat_140 may contain loci that were under selection in N. American soybean breeding programs.

A total of 620 and 42 regions (100 kb windows), contained loci with high average fixation indices (F_st_≥0.6) between *G. soja* and landrace and between landrace and elite populations, respectively (Table 7, Table S2). These regions may contain loci associated with domestication from the wild soybean or with selection imposed during 70 years of North American soybean breeding, respectively. The examination of these regions will provide the basis for much further investigation aimed at the genic content of regions that underwent selection during domestication and during the genetic improvement of soybean adapted to the diverse soybean producing environments of North America. At this time we thought it appropriate to briefly describe one of the genome regions that was apparently under selection during soybean domestication and one with the selection imposed by North American soybean breeding. In the case of a region associated with domestication, we observed that the region flanked by Sat_355 and Satt474 of soybean linkage group B2 [14], i.e., position13,784,007–33,076,661 of the Gm14 in Glyma1.01 [15], has been reported to contain major quantitative trait loci (QTL) controlling seed yield and plant height [16], oil [17], seed size [18]–[20], and stem strength [21]. Most of these traits were very likely affected during domestication, accordingly, we observed that most of the F_st_ values between *G. soja* and the landraces in this region were greater than 0.6. In contrast, most of the F_st_ values between the elite and landrace populations were less than 0.1 (Fig. 2a).

**Table 7:** Number of 100 kb regions across the 20 soybean chromosomes with Fst ≥0.6 in comparisons of *G. soja* vs. landraces and landraces vs. elite cultivars.

Chromosome	Number of regions with Fst ≥0.6 in *G. soja*vs. Landrace	Number of regions with Fst ≥0.6 landrace vs. elite and with 100 kb interval to the adjacent regions
Gm01	48	3
Gm02	21	
Gm03	26	
Gm04	11	11
Gm05	72	
Gm06	17	
Gm07	41	1
Gm08	26	3
Gm09	15	
Gm10	35	
Gm11	32	
Gm12	57	20
Gm13	28	
Gm14	51	
Gm15	2	
Gm16	8	1
Gm17	11	2
Gm18	14	
Gm19	44	
Gm20	61	1
Total	620	42

In the case of selection imposed during the years of North America soybean breeding, an example of a strong peak of F_st_ between the landrace and elite populations was observed between 2.7–5.0 mbp of Gm04 in Glyma1.01 (Fig. 2b). QTL for resistance to soybean cyst nematode (SCN) race 2 [22], [23] seed size [24], pods per nod [25] and seed protein concentration [20] have been reported in the region flanked by Satt396 and Sat_140 of soybean linkage group C1 [14], i.e., position 2,719,766–5,221,447 of Gm04 in Glyma1.01 [15].

## Discussion

A number of options exist for high throughput SNP genotyping and the Illumina GoldenGate platform is a commonly used assay in plants. Examples of its application in plants include 1536-SNP assays in barley [4], [26], maize [27], *Aegilops tauschii* [28], common bean [29], and a 96-SNP assay in wheat [30]. In soybean, a universal linkage panel containing 1536 SNPs for high-throughput soybean QTL mapping was developed [31]. The Illumina Infinium assay provides a significantly higher level of SNP genotyping capacity and recent Infinium assays with successful allele calls for nearly 50,000 SNPs were reported in maize (*Zea mays* L.) [32] and assays for nearly 8,000 SNPs have been reported in sunflower (*Helianthus annuus* L.) [33], apple (*Malus*×*domestica*) [34] and tomato (*Solanum lycopersicum*) [35]. The SoySNP50K is the first Infinium beadchip containing >50 k SNPs in soybean. This assay is being applied to genotype more than 19,000 cultivated and wild soybean accessions in the USDA Soybean Germplasm Collection and more than 960 recombinant inbred lines in each of two mapping populations at the USDA-ARS, Beltsville, MD. The resulting datasets will assist in the application of genome-wide association studies of important traits and to detect signatures of selection and evolution, pinpoint genes or regions controlling phenotypic variants and define whole-genome LD patterns and regions associated with soybean domestication. The SoySNP50K BeadChip will expedite the development of high resolution linkage maps to improve the previous whole genome sequence assembly and to position unanchored scaffolds to the current assembly (Glyma1.01).

The justification for the reduced representation library used in the SNP discovery was to reduce the complexity of the subset of the genome that was sequenced for SNP discovery and to generate large sets of common fragments for alignment. This approach may leave portions of the genome without sequence and thus without SNPs. However, due to the use of 10 different restriction enzymes and the resulting diversity of restriction sites, the identified SNPs were, for the most part, randomly distributed in the soybean genome.

The density of genes and the recombination rate in euchromatic and heterochromatic regions differ dramatically with much lower recombination and a lower gene density in the heterochromatic vs. the euchromatic regions [10]. Low recombination rate would be expected to result in more extensive LD in the heterochromatic regions, although the actual extent is unknown. Thus, we attempted to design a beadchip with higher SNP density in the euchromatic than in the heterochromatic regions i.e., 1 SNP every 9.1 kb in euchromatic regions and one SNP every 49.1 kb in heterochromatic regions. To avoid clustering, SNPs were selected such that the distance between SNPs was as near as possible to 9.1 kb in the euchromatic and 49.1 kb in the heterochromatic regions. Thus, the selected SNPs would evenly cover both the euchromatic and heterochromatic regions. The very different SNP densities in euchromatic and heterochromatic regions and their association with the anticipated difference in the magnitude of LD in the two regions were done in anticipation of the application of genome wide association studies in diverse populations of accessions selected from the USDA Soybean Germplasm Collection.

During the SoySNP50K BeadChip development, account was taken of the factors that were essential for a successful assay design i.e., high allele call success rate, high minor allele frequency and the even distribution of SNPs in heterochromatic and euchromatic regions across the soybean genome. The success rate of a beadchip is subject to the bead type success rate during manufacture and the rate of successful allele calls in genotyping. The bead type success rate is the proportion of the total number of attempted assays present in the final product. Illumina guarantees a bead type success rate of >80% but not 100% due to the imperfect efficiency of manufacturing steps such as synthesizing oligonucleotide probes, attaching probes to silica beads, pooling beads, loading bead pools and decoding the beads identified on the beadchip (http://www.illumina.com/Documents/products/technotes/technote_iselect_design.pdf). The 86% bead type success rate for the SoySNP50K beadchip is in the normal range defined by Illumina, Inc. The allele call success rate is the proportion of the total number of assays generating reliable allele calls among the total number of assays in the final product. Reliability of the SNP allele call is dependent on the intensity of fluorescent signals from both alleles in the SNP Graph displayed by the GenomeStudio software and the distinct separation of the clusters of the allelic classes i.e., the two homozygotes and the heterozygotes, as displayed in the normalized theta output of GenomeStudio. Because the fluorescent signal of SNPs may vary with DNA samples and assays, the incidence of poor allele clustering such as multiple or indistinguishable clusters of samples may occur and lead to inaccurate allele calls. Based on our standard to define SNPs with successful allele calls, the rate of successful allele calls using the SoySNP50K beadchip was 91%, which is comparable to the rate of 77–91% reported in human populations using the Illumina Infinium 550 K beadchip [36], 92% in cattle using Illumina Infinium assay BovineSNP50 [37] and 81% in horse using the Illumina commercial assay EquineSNP50 [38].

Rare alleles can produce spurious associations between SNP markers and phenotypes and are therefore often not used in certain genetic analyses. Therefore, SNPs with minor allele frequency below a certain threshold should be avoided. In order to detect SNPs with high minor allele frequency, we sequenced mixed DNAs from six diverse cultivated and two wild soybean genotypes and were able to identify SNPs by aligning reads from the mixed DNA to the reference sequence. Because the SNPs with one allele identical to the reference allele and another to the alternative allele that was present in at least one of the genotypes as determined using short reads of individual genotypes were given the highest priority score, these SNPs were most likely retained during subsequent selection. Statistically, in the mixed DNA, reads containing the minor allele were less likely to be present than reads containing the common allele. We observed that only 7% of the SNPs assayed with SoySNP50K had a minor allele frequency <0.05 in the 288 *G. max* and *G. soja* accessions analyzed. This proportion is generally lower than that reported in various populations of dog, cattle, horse, pig and sheep using Illumina BeadChips [37], [39], [40].

Numerous candidate regions associated with domestication and selection imposed by soybean breeding in North America were identified by examining the F_st_ between the populations. The example of the high fixation indices (F_st_≥0.6) between *G. soja* and landrace in the 13–30 Mb region on Gm14 (Fig. 2a) provides one such region that is likely the result of a domestication sweep. While the high Fst between the elite cultivars and landraces on Gm04 (Fig. 2b) identifies a region that was likely under selection imposed by North American soybean breeders, it is not immediately clear why this region was the target of strong selection. As indicated earlier, QTL for resistance to soybean cyst nematode (SCN) race 2 was been reported in this region [22], [23]. However, the QTL was present in PI 438498B and was not reported until 2001 and is thus unlikely to have been used in the breeding of the 96 elite cultivars included in this study that were released between 1990 and 2000. Relative to the other QTL reported in this region i.e., seed size [24], pods per node [25] and seed protein concentration [20] there is no suggestion that breeders have specifically targeted this region in any type of marker assisted selection program. However, it is possible that selection for improved agronomic traits and higher yields over many years of N. American soybean breeding has altered allele frequency in this region on Gm04. There are data available in the USDA Germplasm Resources Information Network (GRIN) database for seed size (g/100 seeds) for some of the 96 landrace and 96 elite cultivars we analyzed. Gizlice et al. [41] analyzed the pedigrees of modern N. American soybean cultivars and identified 25 landraces that were the ancestors of modern N. American cultivars that contributed 90% of the genetic base of modern cultivars. Seed size data for 23 of the 25 ancestors were available in GRIN and had a mean seed size of 16.4 g/100 seeds. GRIN also contained data for 39 of the 96 elite cultivars we analyzed. These had a mean seed size of 14.7 g/100 seeds. This suggests that selection over the many years of N. American soybean breeding has reduced seed size. The soybean decade’s study that is comparing soybean ancestors along with cultivars developed over the years supports the contention of reduced seed size of modern cultivars versus ancestral cultivars (Personal communication – R. Nelson). Further analysis of this genome region is clearly of interest.

Knowledge of the positions of the regions associated with domestication and with gene frequency changes associated with N. American soybean breeding is critical for the identification of genes controlling important agronomic traits. However, due to limited sample size, the span of the regions identified in the current study are still quite large. As the analysis of all of the more than 19,000 annual *Glycine* accessions in the USDA Soybean Germplasm Collection with the SoySNP50K proceeds it is anticipated that regions under selection will be detected with high statistical power and resolution. We can anticipate that the identification of such regions with strong signatures of selection will be the targets of investigation with the goal of identifying the impacted phenotype and the gene or genes that underlie the phenotypic change(s).

## Materials and Methods

### Sequencing a Panel of Eight Soybean Genotypes with the Illumina Genome Analyzer

Six soybean genotypes, Essex, Evans, Archer, Minsoy, Noir 1, and Peking and two wild soybean accessions, PI 468916 and PI 479752, were used for re-sequencing. The eight accessions are parents of soybean mapping populations. DNA of each of the eight genotypes was extracted from leaf or root tissues and each was digested separately with two sets of five restriction enzymes: Set 1: HaeIII, PsiI, SspI, RsaI; and Ms1I and Set 2: Cvik1–1, DraI, SnaB1, PshA1, and Stu1. The resulting digests were combined and the restriction fragments were size selected on a 1.0% agarose gel and the 110–140****bp fraction was purified and prepared for Illumina GA sequencing (Illumina Inc, San Diego, CA). Protocols for adapter ligation, cluster generation and sequencing by synthesis on the Illumina GAII were followed and read lengths of 35–45****bp were obtained. The Goat Pipeline (v 1.3) was used for base calling and quality assessment. A total of 56 lanes of sequence data were collected from the eight genotypes, in addition, 15 lanes of sequence data were collected from mixed DNA of Archer, Evans, Noir 1, Minsoy and Peking.

### Sequence Alignment and SNP Identification

The whole genome sequence (WGS) of Williams 82 (Glyma1.01) [10] was used as the alignment reference and the short sequences from Essex, Evans, Archer, Minsoy, Noir 1, Peking, PI468916 and PI479752 were mapped to the reference sequence using MAQ (v0.6.8) [42] and Illumina ELAND software (Illumina Inc, San Diego, CA) of the Goat pipeline. SNPs were called using MAQ and Illumina CASAVA software (Illumina Inc, San Diego, CA). Default parameters of MAQ and CASAVA for SNP identification were applied except for the minimum read depth in MAQ, which was set to 1. Called SNPs were eliminated for Infinium design if any of the following conditions were met: both alleles were present in a genotype (indicative of paralogous sequence); the SNP allele from an individual genotype did not match the ambiguity code or the allele from the mixed DNA; 60 bases of SNP-flanking sequences contained N; there was another SNP residing within 25 bases of an identified SNP; the 25 nt SNP flanking sequences were not specific in the whole genome and the SNP type was A/T or G/C or an indel.

### Initial Verification of SNP Calls from MAQ and CASAVA in Order to Set Parameters to Effectively Select SNPs for Infinium BeadChip Design

In order to optimize the selection criteria to effectively design the Infinium BeadChip, a total of 767 SNP-containing loci were randomly selected for preliminary validation of the accuracy of SNP calls. A PCR primer set targeting the SNP position was designed to each of the loci. Primers were designed using standalone Primer 3 (http://primer3.sourceforge.net/releases.php). The targeted PCR product length ranged from 400–600****bp, with the annealing temperature from 55–62°C and the primer length from 18–27 nucleotides. In order to reduce the likelihood of non-specific annealing of primers, all primer sequences were examined with e-PCR software [43] with the parameters of maximum number of mismatching nucleotides in one primer sequence of N = 3 and maximum number of gaps of one primer sequence of G = 1. Primer sets that were not uniquely aligned to the genome sequence under this stringency were discarded and reselected. The resulting amplification from genomic DNA of the genotypes containing the non-reference allele was sequenced with the ABI 3730 (Applied Biosystems, Foster City, CA) as described by Choi et al. [44], and the sequence was aligned to the soybean reference sequence. Variants were identified with PolyBayes SNP discovery software [45], [46].

### Identification and Screening of Pre-existing SNPs at dbSNP of NCBI

A total of 5300 SNPs in soybean had previously been genetically mapped and assayed in multiple genotypes using Sanger sequencing [9], [31], [44]. The flanking sequences of the SNPs were aligned to the WGS of Williams 82 using standalone Megablast software (http://www.ncbi.nlm.nih.gov/blast/megablast.shtml) and the specific positions of SNPs in the genome were determined with W = 50, cutoff percentage of alignment = 98 and low complexity filtered as described previously [15]. A total of 2739 of the 5300 SNPs passed filters as described above and were included for further selection.

### Procedure and Iteration Algorithm for the Selection of a Core Set of SNPs

SNPs were assigned a priority group of A, B, C or D as follows: A. SNPs were called from at least one genotype with read depth greater than or equal to 1, and the two SNP alleles from the reference and non-reference sequences were consistent with the IUB code called from mixed DNA or the SNPs were already present in dbSNP at NCBI and had previously been successfully assayed in multiple genotypes. B. SNPs were identified in at least two genotypes. C. SNPs were detected in only one genotype but the read depth was greater than 3. D. SNPs were called from only one genotype with read depth less than three, and the non-reference SNP allele was also present in the mixed DNA. A priority score (R_i_) was then calculated for each SNP as the ratio of verified to examined SNPs in each priority group among the 767 SNP-containing loci in the verification of SNP calls using ABI 3730 Sanger sequence analysis. In addition, the Illumina Infinium design score provided by Illumina, which is a predictor of assay success, was assigned for each SNP using Illumina’s Assay Design Tool for Infinium (Illumina, Inc., San Diego). SNPs with a design score less than 0.4 were eliminated. For each SNP, a SNP selection index score (I_i_) was calculated by multiplying the priority score (R_i_) with the Illumina design score (D_i_), i.e. I_i_ = R_i_×D_i_.

Based on the SNP selection index scores, an iteration algorithm was developed to select the desired number of SNPs with desired physical distance between adjacent SNPs in euchromatic and heterochromatic regions and with elevated index scores. The total number of SNPs to be selected from the euchromatic and the heterochromatic regions was determined based upon the ratio of genetic map distance to physical distance in bp in the euchromatic versus the heterochromatic regions. The definition of the physical lengths of the euchromatic and heterochromatic regions was as follows: The physical positions of mapped SNPs and simple sequence repeats (SSRs) [9], [14], [44], [47], [48] in the soybean genome were determined by BLAST analysis of the SNP and SSR-containing source sequence to the soybean whole genome sequence using the standalone Megablast software as previously described [15]. The cumulative genetic distances (cM) [31] were plotted against their cumulative physical distance (Mbp) to determine the base pair/centiMorgan relationship via the common SSR and SNP loci positions on the genetic linkage map and their genome sequence along each chromosome. The region between the two inflection points of the cumulative genetic distance against cumulative physical distance on the plot was defined as the heterochromatic region. The reliability of defining heterochromatic regions using this method was examined in rice, and showed that the heterochromatic region defined by suppressed recombination rate was in agreement with that identified by the conventional DAPI staining method [48]. Since the ratio is about 5∶1, the density of selected SNPs was determined to be 5 times greater in the euchromatic vs. the heterochromatic regions. The desired number of SNPs to be selected from euchromatic and heterochromatic regions of each chromosome was calculated as L_ij_/T_j_×W_j_, where L_ij_ is the sequence length (bp) of the euchromatic (j = 1) or heterochromatic region (j = 2) for the i^th^ chromosome and T_j_ and W_j_ were the total sequence length (bp) and total number of SNPs to be selected in the euchromatic or heterochromatic regions across the 20 soybean chromosomes, respectively. For each iteration of the algorithm, gaps (sequence length) between adjacent SNPs were calculated along each chromosome. Subsequently, the smallest gap along each chromosome was identified and the SNP selection index scores of the SNPs flanking the gap were calculated. The SNP with the smallest SNP selection index score was removed. If the SNP selection index scores of the two SNPs were identical, the SNP with the smallest gap to another adjacent SNP was eliminated. If two or more gaps in a single iteration were identical, the SNP with the smallest SNP selection index score among the SNPs flanking the gaps was eliminated. The iteration continued until the desired number of SNPs in euchromatic and heterochromatic regions was reached. In addition, the whole genome sequence of Williams 82 (Glyma1.01) contains 236 unanchored scaffolds with lengths from 10****kb to 100 kb and 51 unanchored scaffolds with lengths greater than 100****kb. SNPs identified in these scaffolds were also included in order to facilitate anchoring of these scaffolds for the next genome assembly.

### Validation of the SoySNP50K iSelect SNP Beadchip

A group of 96 diverse landraces, 96 elite cultivars and 96 *G. soja* accessions were used to determine the allele frequency of selected SNPs on the SoySNP50K iSelect SNP BeadChip. The 96 landraces [49] were from China, Japan and Korea and represent a wide range of geographic origin and maturity groups. The 96 elite cultivars were from North America and represent a diversity of publically developed cultivars released from 1990 to 2000 [31], [50]. The 96 *G. soja* accessions were from China, Korea, Japan and Russia and were selected based upon the wide ranges of latitude and longitude from where they were collected (Table S3). SNP genotyping was conducted on the Illumina platform by following the Infinium® HD Assay Ultra Protocol (Illumina, Inc. San Diego, CA). The Infinium II assay protocol includes the procedures to make, incubate and fragment amplified DNA, prepare the bead assay, hybridize samples to the bead assay, extend and stain samples, and image the bead assay. The SNP alleles were called using the GenomeStudio Genotyping Module v1.8.4 (Illumina, Inc. San Diego, CA). A stringent standard to define SNPs with successful calls was adopted so that only those SNPs with two or three discrete clusters and both alleles with high signal intensity on the SNP Graph Alt based on the assessment populations were counted and included for the calculation of the success rate of genotyping even though some of the exclusions were likely real SNPs.

### Statistical Analysis

A Pearson’s correlation coefficient was used to investigate the correlation between the SNP selection index score, the Illumina Infinium SNP design score and the rate of SNPs with successful allele calls.

Genetic distance among pairs of genotypes within the 96 landraces, within the 96 elite cultivars and within the 96 wild accessions was calculated as the ratio of the number of polymorphic SNPs vs. total number of SNPs for which allele calls were made for the pair.

The fixation index (F_st_) is a measure of population differentiation. F_st_ values range from 0 to 1, with zero representing no allele differentiation and 1 representing complete allele differentiation between the two populations. The F_st_ between *G*. *soja* and the landraces and between the landraces and elite cultivars were calculated for each locus using the software Arlequin v3.1 and the significance of the F_st_ was tested using a non-parametric permutation approach [51]. Subsequently, an average F_st_ of SNP loci in 100****kb windows along each chromosome was calculated.

The physical position of SSR markers flanking QTL controlling seed size, protein and oil content, seed hardness, seed color, and other traits that had been previously reported were obtained from BARCSOYSSR_1.0 [15].

## Supporting Information

Table S1 **SNP type, chromosome and SNP position, alternative alleles, SNP flanking sequences, priority group, scores, polymorphism, position in gene (if applicable), MAF in elite, landrace and wild soybean populations.**
(XLSX)

Table S2 **Average F_st_** ***G. soja*** **vs. Landraces and F_st_ Elite cultivars vs. Landraces in 100 kbp windows along each chromosome.**
(XLSX)

Table S3 **Accession IDs, province and country of origin, and latitude and longitude at which the 96** ***G. soja*** **accessions used in this study were collected.**
(XLSX)
